# TRPV4 receptor as a functional sensory molecule in bladder urothelium: Stretch‐independent, tissue‐specific actions and pathological implications

**DOI:** 10.1096/fj.201900961RR

**Published:** 2019-11-21

**Authors:** Max W. G. Roberts, Guiping Sui, Rui Wu, Weifang Rong, Scott Wildman, Bruce Montgomery, Ahmed Ali, Steve Langley, Michael R. Ruggieri, Changhao Wu

**Affiliations:** ^1^ School of Biosciences and Medicine University of Surrey Guildford UK; ^2^ Guy's and St Thomas Hospitals NHS Trust London UK; ^3^ University Hospitals Coventry and Warwickshire NHS Trust Coventry UK; ^4^ Department of Physiology Shanghai Jiaotong University School of Medicine Shanghai China; ^5^ School of Pharmacy University of Kent Chatham UK; ^6^ Frimley Health NHS Trust Frimley UK; ^7^ Royal Surrey County NHS Trust Guildford UK; ^8^ Department of Anatomy and Cell Biology Temple University Philadelphia USA

**Keywords:** aging, ATP release, overactive bladders, TRPV4 receptor, urothelium

## Abstract

The newly recognized sensory role of bladder urothelium has generated intense interest in identifying its novel sensory molecules. Sensory receptor TRPV4 may serve such function. However, specific and physiologically relevant tissue actions of TRPV4, stretch‐independent responses, and underlying mechanisms are unknown and its role in human conditions has not been examined. Here we showed TRPV4 expression in guinea‐pig urothelium, suburothelium, and bladder smooth muscle, with urothelial predominance. Selective TRPV4 activation without stretch evoked significant ATP release—key urothelial sensory process, from live mucosa tissue, full‐thickness bladder but not smooth muscle, and sustained muscle contractions. ATP release was mediated by Ca^2+^‐dependent, pannexin/connexin‐conductive pathway involving protein tyrosine kinase, but independent from vesicular transport and chloride channels. TRPV4 activation generated greater Ca^2+^ rise than purinergic activation in urothelial cells. There was intrinsic TRPV4 activity without exogeneous stimulus, causing ATP release. TRPV4 contributed to 50% stretch‐induced ATP release. TRPV4 activation also triggered superoxide release. TRPV4 expression was increased with aging. Human bladder mucosa presented similarities to guinea pigs. Overactive bladders exhibited greater TRPV4‐induced ATP release with age dependence. These data provide the first evidence in humans for the key functional role of TRPV4 in urothelium with specific mechanisms and identify TRPV4 up‐regulation in aging and overactive bladders.

**Abbreviations**
DSM: detrusor smooth muscle
EET: epoxyeicosatrienoic acids
FPH: Frimley Park Hospital
FT: full thickness
GP: guinea pig
GSK: GSK1016790A
HC: HC‐067047
IDO: idiopathic detrusor overactivity
IHC: Immunohistochemistry
MMH: Medway Maritime Hospital
OAB: overactive bladder
PMC: pontine micturition center
RBL: rat brain lysate
RSCH: Royal Surrey County Hospital
SFK: Src‐family tyrosine kinases
TRPV: transient receptor potential vanilloid

## 1 INTRODUCTION

Age‐related diseases present a huge societal challenge to our aging society. One class of highly prevalent, costly but under‐studied debilitating chronic aging consequences is bladder disorders such as overactive bladder and recurrent bladder infections. Bladder diseases have a prevalence of 15%‐20% rising to 40% over the age of 65 years, seriously reduce the quality of life and are the main reasons for entering into care‐homes. Specific treatment is currently not available due to poor understanding of their pathogenesis.

The urothelium, the inner epithelial lining of the urinary bladder, has recently been recognized as a new sensory structure in addition to its classic barrier function.^1^ Release of ATP from the urothelium in response to the physiological stimulus of bladder distension,^2^ expression of purinergic receptors (P2X3) on sensory nerve ending beneath the urothelium,^3^ and the ability of ATP to trigger afferent firing^4^, ^5^ provide experimental evidence for this tissue layer to function as a sensory tissue, with ATP as the sensory mediator. P2X3 deletion‐induced suppression of the micturition reflex and afferent firing^3^, ^5^ gives further weight to its physiological relevance. Expression of vanilloid receptors on the urothelium^6^ and the effect of TRPV1 deletion on bladder voiding^7^ suggest that this tissue can respond to noxious stimuli such as high temperature and acidic pH. Changes to the urothelium in pathological animal models^8^, ^9^, ^10^, ^11^ and human bladder pathologies^12^, ^13^, ^14^ emphasize a role of this structure in pathogenesis of bladder dysfunction.

A sensory role of the urothelium is further evidenced by expression of a variety of receptors typically expressed in sensory neurones, and nociceptive and mechanoreceptive cells.^9^, ^15^, ^16^, ^17^, ^18^, ^19^, ^20^, ^21^, ^22^, ^23^ These “sensory molecules” may potentially enable the urothelium to respond to a range of physiological and pathological stimuli.^1^ Experimental proof of these molecules functioning as sensory molecules will greatly enhance our understanding of urothelial sensory function. Some purinoreceptors and muscarinic receptors on the urothelium have been shown to function as sensory receptors for physiological signals.^24^ Most of the other receptors, however in particular, those receptors that sense pathological stimuli, have not yet been adequately explored. Identification of a pathological sensory molecule will be a major step forward in understanding the pathological role of the urothelium.

One such potential sensory molecule is the transient receptor potential vanilloid 4 (TRPV4) channel, a TRP superfamily non‐selective cation channel preferentially permeable to Ca^2+^, a major second messenger.^25^, ^26^ TRPV4 is commonly considered to be mechano‐ or osmo‐sensor^27^, ^28^, ^29^, ^30^ and can also be activated by moderate heat.^26^, ^31^ This indicates significant constitutive activity at physiological temperatures and an up‐regulation in fever and local temperature rise. Certain chemical stimuli are also able to activate TRPV4.^25^, ^32^, ^33^, ^34^ As many of the above factors are encountered during inflammatory conditions, TRPV4 will respond to inflammation and hence acts as pathological sensor.

TRPV4 expression has been demonstrated by immunostaining in urothelium from mouse^35^, ^36^ and rat.^35^ TRPV4‐positive staining was also reported in human urothelial umbrella cells, albeit from limited samples.^37^ However, the tissue distribution of TRPV4 in the bladder wall, their molecular identification and quantification remain to be determined.

The function of TRPV4 was first shown in cultured urothelial cells as a Ca^2+^ rise in response to 4α‐phorbol 12,13‐didecanoate (4α‐PDD), an early non‐specific TRPV4 agonist,^20^, ^35^, ^38^ not selective for TRPV4.^39^ 4α‐PDD also increased ATP release from these cultured urothelial cells.^38^, ^40^ Hypotonic cell swelling/stretch‐induced Ca^2+^ rise and ATP release from the cultured urothelial cells has been suggested to be due to TRPV4 activity in these studies. These previous studies focused mainly on cultured urothelial cells which are subject to phenotypic changes,^41^ and stretch as a method of investigating TRPV4, which activates many stretch‐sensitive but TRPV4‐independent processes, and have not used selective TRPV4 activator and antagonist.^39^, ^42^, ^43^ Gevaert et al^35^ further reported that TRPV4‐deficient mice exhibited a decreased spontaneous activity in isolated bladder strips, and decreased stretch‐induced ATP release in isolated whole bladders, although measurement of ATP release directly from the urothelium layer was not performed. A major step forward in TRPV4 physiology awaits clarification of the mode of action for TRPV4 in native urothelium and smooth muscle, preferably in multicellular preparations to preserve the functional syncytium^10^ and in response to stretch and stretch‐independent stimuli which has been little studied. More importantly, the functional role of TRPV4 in human conditions has not been explored, prompting urgent need for this translational investigation.

The objectives of this study are first to characterize the properties of TRPV4 and its contribution to urothelial and smooth muscle activation, through specific activation of the receptor in native animal bladder tissue, and the underlying subcellular mechanism and, second, to demonstrate its relevance to human conditions and finally to investigate changes to this receptor in aging and pathology. The data from this study provide crucial evidence for the physiological importance and pathological implications for this receptor in the bladder with mechanistic insight.

## 2 MATERIALS AND METHODS

### 2.1 Animal and human tissue preparations

Young (2‐5 months) and aging (24‐36 months) male Dunkin‐Hartley guinea pigs (GPs, B&K Universal) were maintained under standard feeding conditions and used for experiments. GPs between 2 and 5 months old are mature young adults, capable of breeding and are widely used in laboratory studies. From the age of 18 months, GPs begin to exhibit age‐related structural and functional changes in both general and bladder‐specific physiology and therefore represent early old age.^44^, ^45^, ^46^ Qualitative age‐related urinary tract changes in humans, such as increased prostate growth, begin to emerge in GPs from 2 years and thereafter, indicating GPs above this age as a representative model for translational age‐related studies.^47^, ^48^ Animals were humanely culled by a schedule 1 procedure in accordance with the United Kingdom Animals (Scientific Procedures) Act of 1986. All experiments were approved by the local ethics committee and followed UK home office regulations.

The urinary bladders were removed quickly dissected under microscopic guidance as previously described.^49^ To determine tissue layer‐specific action, “full thickness” (FT), “smooth muscle only,” and “mucosal” strips were used. The mucosa (urothelium and suburothelium) was gently separated from underlying tissue by careful microdissection along the natural cleavage plane.

Human mucosal tissue biopsies were obtained by cystoscopy from bladders of patients undergoing diagnostic endoscopy. Samples were collected with informed patient consent and the approval of the National Research Ethics Committee (South East Coast—Surrey) in accordance with the Helsinki declaration. In patients with suspected tumors or cancer, bladder samples were taken from macroscopically normal areas. Samples from patients with no symptoms of OAB (non‐OAB cohort, age 72 ± 2.3) and those from patients with symptoms of OAB (clinical diagnosis, frequency ≥ 8/day, urgency with or without urgency incontinence), diagnosed with idiopathic detrusor overactivity (IDO, OAB with no neurological abnormality, OAB cohort, age 60 ± 5.0) were collected in the same way from Frimley Park Hospital (FPH), the Royal Surrey County Hospital (RSCH), and Medway Maritime Hospital (MMH). These mucosal biopsies were treated similarly to GP tissue and cut into longitudinal strips for experimentation.

Bladder strips were mounted within a customized horizontal Perspex perfusion trough/organ bath with one end of the tissue tied to a fixed hook and the other to an isometric tension transducer with micromanipulator, allowing precise orientation of the tissue.^49^ Mounting of strips was performed under microscopic guidance to ensure accuracy and avoid accidental stretch and damage, which can generate ATP release. A water‐jacket system was used to maintain experimental solutions and tissue at 37°C. The tissue preparations were superfused with HEPES‐buffered physiological Tyrode's solution (pH 7.4, 37°C) at a resting rate of 2 mL min^‐1^ for at least 45 minutes prior to experimentation to allow full tissue stabilization. Both ATP release and contractile activity of tissue strips were measured (*see below*).

Isolated mucosal tissue was also used to prepare single urothelial cells by enzymatic dissociation for the purpose of intracellular Ca^2+^ measurement. Cells were dissociated using a collagenase type‐1 (1.0 mg/mL Worthington Biochemical Corp, USA)‐based enzyme mix dissolved in HEPES‐buffered Ca^2+^‐free Tyrode's solution.^24^

### 2.2 Solutions

The HEPES‐buffered Tyrode's solution used for preparation, incubation, and superfusion of tissue during experiments contained (in mM) 132 NaCl, 4 KCl, 1.8 CaCl_2_, 1.0 MgCl_2_, 0.4 NaH_2_PO_4_, 10 HEPES, 6.1 glucose, and 5 Na pyruvate (adjusted pH 7.4, osmolarity 311.2 mosM). For tissue isolation and storage, a nominally Ca^2+^‐free modified Tyrode's solution was used, prepared as above with the omission of 1.8 mM CaCl_2_ and replaced by 1.8 mM MgCl_2_ to maintain the divalent cation concentration. For cell dissociation, the following were added; collagenase type I (1.0 mg/mL, Worthington biochemical, Lakewood, NJ), hyaluronidase type I‐S (0.25 mg/mL) and type III (0.25 mg/mL), trypsin inhibitor type II‐S (0.45 mg/mL), and BSA (2.5 mg/mL). For Ca^2+^‐free modified Tyrode's solution, the CaCl_2_ was substituted with additional MgCl_2_ (total MgCl_2_: 2.8 mM) with the addition of EGTA (0.1 mM, [Sigma, UK]) to buffer any residual calcium to pCa of 8. The drugs and compounds used for interventions during physiological experiments were dissolved in either ultra‐pure water or DMSO (BDH, UK) and stored at −20°C as stock solutions of at least 1000 times their experimental concentrations, before final dilution on the experimental day. The experimental concentration of agonists was chosen based on preliminary work demonstrating a reproducible effect without causing significant desensitization, being above the EC_50_ but below the maximal effect. Experimental concentrations of antagonists were calculated based on their affinity for receptors for predominant blockade, and where appropriate, to oppose the concentration of same‐receptor agonist used, such that >95% of receptors were inhibited. RIPA lysis buffer for western blotting contained final concentrations (in mM) 50 Tris‐HCl, 150 NaCl, 2 ethylene diamine‐tetraacetic acid (EDTA), 50 NaF, 1% NP‐40, 1% sodium deoxycholate, protease inhibitor cocktail (“cOmplete, Mini, EDTA free PI cocktail” Roche, UK) in “ultra‐pure” distilled water, pH 7.5 ± 0.05. All chemicals were from Sigma unless otherwise stated.

### 2.3 Measurement of ATP

Once mounted within the perfusion trough, tissue strips were left to stabilize in Tyrode's solution for 45 minutes prior to experimentation.^49^ Superfusate samples were collected from the sampling point (in close vicinity of the mucosa, 2/3rds downstream of the length of the tissue) using a micropipette tip under a microscope and immediately evaluated for ATP content. Samples were taken during the pre‐ and post‐control periods and during the drug intervention at 1, 3, and 5‐min for short observations, followed by sampling at 5‐min intervals thereafter for longer experiments. Mean ATP release before and after the drug intervention was taken as the baseline ATP release. Mean ATP release across all sampling times during the drug intervention was taken as drug effect and expressed as a percentage difference from the baseline. Appropriate controls were carried out for solvents used. For stretch experiments, tissue was mechanically stretched to 150% of the original length for 30 s before the tissue was relaxed to its resting length. These parameters were chosen based on preliminary work that demonstrated a significant and reproducible stretch‐induced ATP release. Superfusate was sampled at 30 s, 1 minutes, 2 minutes, and 3 minutes after initial stretch. Sampled superfusate was immediately assessed for ATP content using the ATP‐dependent bioluminescent “Luciferin‐Luciferase” assay (Sigma, UK).^49^ Luminescence intensity was read using an LKB Wallac 1250 luminometer. Appropriate control readings of reagents and chemicals were also taken to account for any background luminescence.

### 2.4 Measurement of isometric tension

For appropriate interventions, the magnitude of contraction elicited by an agonist was determined. Tissue strips were mounted within the perfusion trough and tied to a force‐displacement transducer coupled with a bridge‐amplifier and isometric tension measured and recorded as described before.^10^ As with ATP measurement, tissue preparations were continuously superfused with HEPES‐buffered Tyrode's solution and drug interventions were introduced via this superfusate.

### 2.5 Measurement of intracellular Ca^2+^

Intracellular Ca^2+^ concentration ([Ca^2+^]_i_) was measured by epifluorescence microscopy using the fluorescent Ca^2+^ indicator dye Fura‐2 as described previously.^49^ The cell suspension was placed in a Perspex perfusion dish mounted on the stage of an inverted microscope maintained at 37°C. After attachment, cells were superfused with a continual flow of oxygenated Tyrode's solution at approximately 2 mL/min^−1^. Cells were then visualized using the microscope and the cell of interest was alternately illuminated with 340 nm and 380 nm excitation wavelengths at 50 Hz. Emitted light between 410 nm and 510 nm split and selected by dichroic mirrors and filters was then collected by a PMT tube/CCD camera. Single cells were challenged with 100 μM ATP or 1 μM GSK1016790A. The ratio of the emitted fluorescence intensities at these two excitation wavelengths was converted into intracellular [Ca^2+^] values using a previously described in vitro calibration method.^50^, ^51^

### 2.6 Immunohistochemistry

Sections of full bladder wall were embedded in OCT (Bright Instrument LTD, UK), snap‐frozen in liquid nitrogen (LqN) and cut into 10 μm thick sections through the transverse plane using a precooled cryostat (Hyrax C25, Zeiss, Germany [−22°C]), before being immediately mounted onto poly‐L‐lysine coated slides. Each section was viewed under a light microscope to ensure structural integrity and presence of all tissue types. Cryosections were fixed in precooled methanol for 5 minutes at −20°C, before phosphate‐buffered saline (PBS, Oxoid) washes. Sections were blocked with 1%BSA/PBS solution for 1 hour at room temperate and incubated over‐night at 4°C in rabbit anti‐TRPV4 polyclonal primary antibody (2.0 μg/mL, Alomone, Israel [AC‐034]; 1:200). Bound primary antibody was detected with Alexa 568‐conjugated Goat anti‐Rabbit IgG (Thermo Fisher Scientific [A‐11011]). A nuclear stain (TO‐PRO3 [Molecular Probes, Netherlands]) was used to aid with visualization of tissue morphology. For dual staining, TRPV4 (Abcam, 1:500) and cytokeratin 18 primary (mouse monoclonal, Sc‐51582, Santa Cruz, 1:200) antibodies were used to show urothelial co‐localization and TRPV4 and ɑ‐smooth muscle actin (mouse monoclonal, A‐2547 Sigma, 1:100) primary antibodies used for smooth muscle co‐localization; secondary antibody: Alexa 488‐conjugated donkey anti‐mouse IgG (A21202, Thermo Fisher Scientific, 1:1000). Stained sections were visualized at 40× magnification (oil‐emersion lens) using a Zeiss LSM510 META laser scanning confocal microscope and images were subsequently analyzed using Axovision LSM 510 Meta software. Appropriate filters and light paths were employed to separate the transmitted excitation light from the emission light, omit any autofluorescence and detect only the desired emission wavelength of each specific antibody/stain (alexa‐568nm [emission 603], TO‐PRO3 642 nm [emission 661]). Appropriate primary antibody omission and peptide antigen pre‐absorption controls were performed to confirm antibody specificity. For each animal bladder investigated (“n number”), three positively stained sections were examined to confirm expression of TRPV4 throughout that bladder, alongside a secondary control section. TRPV4 fluorescence was quantified for each image using ImageJ software (version 2, NIH, USA). An area of interest was defined using the *oval* tool (consistent area of 435.29 units) and this was used to measure the mean pixel density of three different areas of each tissue type within any given image. The mean pixel density for secondary controls was subtracted from positively stain sections to provide a final value for pixel density, representing the strength of TRPV4 staining, allowing relative expression to be compared between tissues.

### 2.7 Western Blot

Separated tissue samples (“mucosal” or “smooth muscle”) were snap‐frozen and homogenized in liquid nitrogen (LqN) using a pre‐cooled pestle and mortar. The homogenate powder was lysed in an appropriate volume of RIPA buffer and sonicated at 100Amp using a VC130PB ultrasonic processor (Sonics & Materials Inc, USA) for 30 seconds to release intracellular components. Samples were centrifuged (100 g, 2 minutes, 4°C) and the protein concentration of supernatants determined using a colorimetric protein assay kit (DC protein assay, BioRad, UK). Supernatants were then mixed in a 1:1 ratio with Tris‐glycine‐SDS sample buffer (Novex, Thermo Fisher, USA) and incubated at 95°C for 5 minutes on a hot plate to fully resolve the lysate. Whole rat brain lysate (RBL) (10 μg) was used as positive control for antibody binding. Tissue lysates (30 μg) were loaded and electrophoretically resolved on precast gels (8%‐16% Mini‐PROTEAN TGX, BioRad, UK) and then transferred to a 0.45 μm nitrocellulose membrane (Novex, Thermo Fisher, USA) using a BioRad blotting system. Membranes were probed with primary antibodies for 1 hour at room temperature and the bound antibody detected using IRDye‐conjugated secondary antibody (LI‐COR Biosciences, Germany). The signal was detected and analyzed using an Odyssey CLx infrared imaging system (LI‐COR, Germany). Each membrane was probed for β‐actin (New England Biolabs, USA) to serve as a loading control. Two anti‐TRPV4 antibodies were tested (Alomone Labs, Israel, 1:200; Abcam, UK, 1:1000).

### 2.8 Lucigenin chemiluminecence

Superoxide (O_2_^−^) production by bladder tissue lysates or whole‐tissue pieces was measured using lucigenin‐enhanced chemiluminescence. For whole‐tissue lysates, FT bladder strips were incubated in either Tyrode's or Tyrode's with GSK (1 μM) for 30 minutes at 37°C. Strips were snap frozen and processed into tissue lysates in the same way as western blotting (LqN homogenization and sonication), in a modified Hanks' Balanced Salt solution (0.8 mM MgCl_2_, 1.8 mM CaCl_2_). Protein concentrations (2‐20 μg/μL) of each sample were determined using a Bradford‐based protein assay kit (Bradford Assay dye, BioRad, UK). For whole‐tissue pieces, freshly obtained bladders were dissected into small (1‐3 mg) pieces of separated mucosa, smooth muscle or FT tissue. On the day of experiment, either a whole‐tissue piece or 25 μL of tissue lysate was transferred to an opaque 96‐well plate with 5 μM lucigenin containing buffer and the basal luminescence measured by a luminometer (BMG Lumistar, Germany). The substrate NADPH (25 μM) was then added and sample luminescence recorded. The cell‐permeable O_2_^−^ scavenger Tiron (10 mM) was then added and luminescence measured for a final time. Sample luminescence was corrected to protein concentration and O_2_^−^ production expressed as median arbitrary light units per minute (MLU/min).

### 2.9 Data analysis

Data are expressed as median ± interquartile range (int. range), or mean ± SEM, where n denotes the number of bladders assessed, unless otherwise stated. All data are normalized to tissue mass or protein concentration unless otherwise stated. All data have been tested for normality using D'Agostino‐Pearson omnibus test. For non‐parametric data, Mann‐Whitney test (unpaired, non‐parametric Student's *t* test) was used to examine two unpaired data sets of unknown distribution. A Wilcoxon matched‐pairs signed rank test was used to analyze paired data sets, for example comparing two different interventions performed on the same bladder strip. A Spearman's rank correlation was used to test the association between two variables. For normally distributed data sets, parametric tests were used to test the difference between two data sets (*t* tests) and among multiple data sets (ANOVA and post‐hoc pair‐wise comparisons). The null hypothesis was rejected at *P* < .05, with the level of significance indicated by *P < .05, **P < .01, and ***P < .001. All statistics were performed using raw data, unless comparing the relative effects of two different interventions. However, graphs display normalized data for clear comparisons. All statistical analysis was performed using Microsoft Excel (2010, version 14.1.6) and GraphPad Prism (2015, version 6.07, GraphPad Software).

## 3 RESULTS

### 3.1 Protein expression of TRPV4 in guinea‐pig bladder tissues

Evidence for the expression of TRPV4 receptors was first sought in ex vivo bladder tissues. Immunofluorescence staining qualitatively demonstrated the existence of TRPV4 receptors and its distribution in different tissue layers in the bladder wall. Western blot measurement determined the specific molecular weight band of TRPV4 proteins and its quantification.

#### 3.1.1 Immunofluorescence

**Figure 1:**
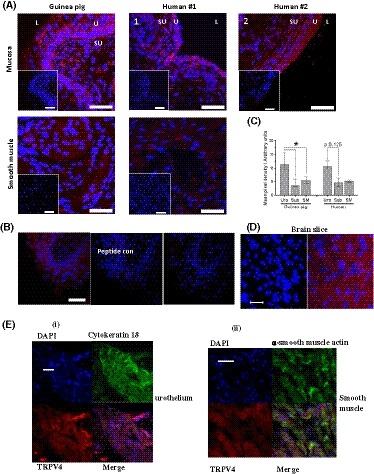
Immunohistochemical localization of TRPV4 in GP bladder tissues. A, Representative images of TRPV4 (Alomome) IHC in GP and human cryosections. TRPV4 fluorescence (red) was detected in both GP and human mucosa and smooth muscle tissue; Insets: control using only the secondary antibodies without TRPV4 primary antibody (anti‐rabbit IgG Alexa 568, life technologies). Nuclei are stained with TO‐PRO3 (Cy5; blue; Invitrogen). U, urothelium; SU, suburothelium; L, lumen. Scale bars represent 50 µm in all images. B, Representative peptide control for Alomone anti‐TRPV4 primary antibody. C, Quantitative analysis of TRPV4 fluorescence. Similar expression patterns observed in both species, with highest fluorescence in the urothelium. Median values [25%, 75%], GP urothelium and suburothelium n = 10, smooth muscle n = 7; human urothelium and suburothelium n = 4, smooth muscle n = 3, *P < .05, Wilcoxon's. D, Cerebral cortex slice TRPV4 positive control, left control with no primary Ab, right TRPV4 staining; E, Co‐staining of TRPV4 with cytokeratin 18 and ɑ‐smooth muscle actin in urothelium (i) and smooth muscle layer (ii)

GP bladders showed TRPV4 immunoreactivity throughout, with strongest expression in the urothelium (n = 10) and lower, similar levels in both the suburothelium (n‐10) and DSM (n = 7, Figure 1A). Specificity of the antibody to TRPV4 was confirmed by performing blocking peptide pre‐absorption controls on four of the GP bladders assessed (one representative image shown, Figure 1B). TRPV4 immunoreactivity was quantified for each tissue and expressed as mean pixel density (Figure 1C). Primary antibody omission controls (representatives shown in figures) were performed to determine the level of background from non‐specific secondary antibody binding. Positive control of brain tissue was also presented (1D). TRPV4 immunoreactivity was significantly greater in urothelium than in suburothelium and DSM. Co‐staining of cytokeratin 18 (urothelium) and ɑ‐smooth muscle actin (smooth muscle) with TRPV4 confirmed TRPV4 expressions in the urothelial layer and smooth muscle layer (1E). These data show that native bladder tissues express abundant TRPV4 receptors across the bladder wall with the highest density in the urothelium.

#### 3.1.2 Western blot

**Figure 2:**
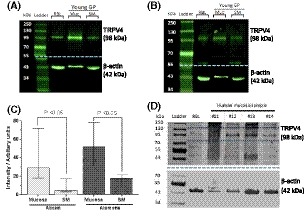
Expression levels of TRPV4. A, GP tissue; Representative western blot of TRPV4 (98 kDa), *Abcam* primary antibody. B, GP tissue; Representative blot of TRPV4 (98 kDa), *Alomone* primary antibody. β‐actin loading controls (42 kDa) shown in blot below dotted blue line. (RBL: rat brain lysate Muc: mucosa, SM: smooth muscle). Membrane was cut at 55 kDa (dashed blue line) and each half probed with the appropriate antibodies. C, Quantified data of TRPV4 expression by densitometry, normalized to its loading control. Samples duplicated and averaged. Results for each tissue type are similar when using either primary antibody; mucosal lysates have significantly higher TRPV4 expression than in smooth muscle lysates with either primary antibody. No significant difference between antibodies for either tissue type. Median values [25, 75], Abcam; n = 5, Alomone; n = 8, Mann‐Whitney. D, Western blot of human mucosal biopsies. Three of the four samples assessed have a positive band at 98 kDa confirming presence of TRPV4. One sample is negative for TRPV4; however, this is likely due to sample degradation due to reuse

To complement the immunostaining data, Western blot was used to identify the specific band for the TRPV4 protein molecule and determine its quantity. TRPV4 expression was detected in separated GP mucosal and smooth muscle lysates, run against RBL positive control tissue. Two anti‐TRPV4 Abs were used to corroborate results and confirm reliability (Alomone, Israel and Abcam, UK). For both antibodies, a single band was observed close to the predicted molecular weight of TRPV4 (98 kDa), between 95 and 100 kDa, for both RBL and bladder tissue lysates (Figure 2A,B). Quantification by densitometry revealed a significantly higher expression of TRPV4 in mucosal tissue than that in smooth muscle when using either Ab (Figure 2C). Mucosal TRPV4 expression was approximately sixfold higher compared to smooth muscle when probed with the Abcam antibody and fourfold higher with the Alomone antibody. The results obtained using one primary antibody were not significantly different from the other, indicating strong reliability. The data show specific TRPV4 protein expression in bladder tissues and significantly higher levels of TRPV4 expression in the urothelial tissue.

### 3.2 Effect of TRPV4 activation on ATP release and contractile activity in the bladder

The evidence for the function of TRPV4 receptors and the mode of action was examined next. Two key physiological functional outputs, ATP release from the urothelial layer and the contraction of the smooth muscle, were examined in different ex vivo bladder tissue preparations by selective activation of TRPV4 with agonist GSK1016790A (GSK).

#### 3.2.1 Effect of activating TRPV4 receptor on bladder functional outputs

**Figure 3:**
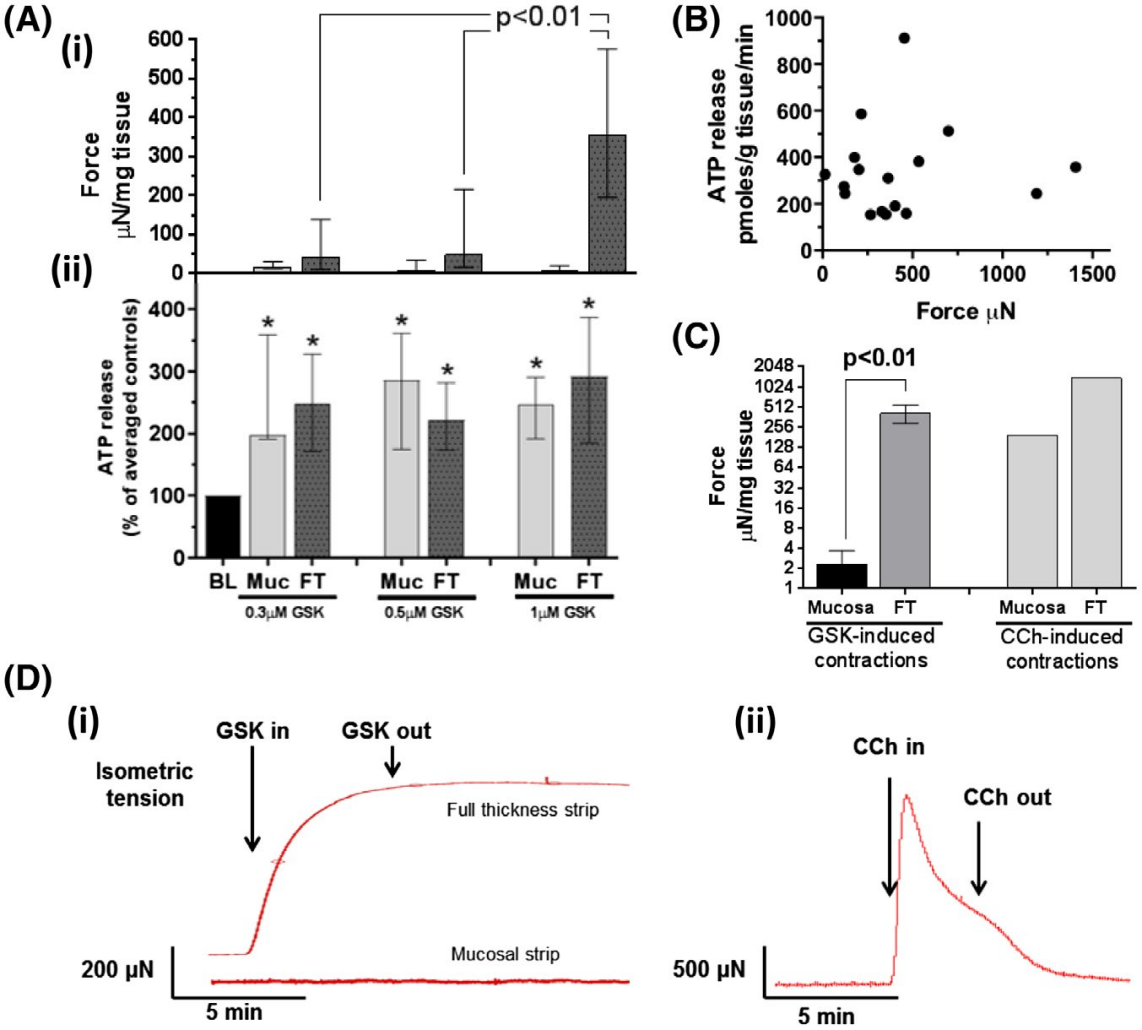
Effects of GSK‐induced TRPV4 activation on GP bladder functional outputs. A(i), Median force of mucosal and full‐thickness contractions generated by various GSK concentrations. Significantly stronger contractions evoked by 1 μM. A(ii), Corresponding median ATP release from same bladder strips. All concentrations significantly increased—ATP release (0.3 μM n = 10; 0.5 μM n = 8; 1 μM n = 18); control: 107 ± 39 pmoles/g tissue/min. B, Relationship between GSK1016790A‐induced contractions and ATP release. No correlation observed between the contraction and level of ATP release in GP FT strips. Spearman correlation coefficient *r_s_* of 0.02374, n = 18. C, Median values of GSK‐induced contractions (n = 8), with record of single 50 μM carbachol (CCh) contraction for reference (n = 1) (note Log2 *y* axis). D, Record of sustained contraction induced by 1 μM GSK in FT strip (i) (and mucosal strip) and transient CCh‐induced contraction in FT strip. Median values [25%, 75%]. A(ii), drug intervention vs. control (basal levels [BL]) compared using Wilcoxon's, *P < .05; All graphs: data sets compared using Mann‐Whitney

The role of TRPV4 receptor in ATP release and contractile function of the bladder was assessed by three concentrations of GSK (0.3, 0.5, and 1 μM) (Figure 3A). GSK application consistently evoked significant contractions in bladder tissue preparations. 1 μM GSK produced significantly stronger and more reproducible contractions in FT strips than lower concentrations (proportion of successfully evoked contractions; 0.3 μM—20%, 0.5 μM—25%, 1 μM—90%, Figure 3A(i)). Mucosal contractions were not observed at any dose. All doses evoked significant ATP release from separated mucosal and FT strips, with no significant difference between any (Figure 3A(ii)). As 1 μM GSK is the only tested concentration capable of evoking both reproducible ATP release and contractions in bladder strips, and is far below that which may have effects on other receptors, this concentration was used for subsequent functional experiments. The force of 1 µM GSK‐induced bladder contractions did not coincide with the level of ATP release as demonstrated in Figure 3B, with a correlation coefficient *r_s_* value of 0.02374. This suggests that TRPV4‐activated urothelial ATP release and smooth muscle contractions from the bladder are independent processes and their down‐stream pathways are also different.

Contractions evoked by GSK (1 μM) in FT strips were of similar magnitude to those evoked by the major physiological neurotransmitter cholinergic agonist carbachol (50 μM). The specificity of muscarinic activation by carbachol was confirmed in our control experiment by the complete block by muscarinic antagonist atropine and no effect of the sodium channel blocker TTX which excludes any measurable contribution from possible TTX sensitive action potential induced neuronal release of acetylcholine under these conditions. However, GSK was not capable of evoking contractions in mucosal sheets, whereas carbachol was able to cause the contraction 24 (Figure 3C). Figure 3D shows the representative TRPV4‐induced contractile response in both mucosa and FT strips next to a typical carbachol trace. GSK‐induced contractions in FT are sustained despite continuous perfusion washout, unlike transient carbachol‐induced contractions. The results suggest that TRPV4 receptors are capable of generating strong muscle contractions in the bladder, but the mode of action is different from the major physiological receptor—muscarinic receptors.

### 3.3 Mode of action and underlying mechanisms for TRPV4 activation

#### 3.3.1 Tissue origin of ATP release

**Figure 4:**
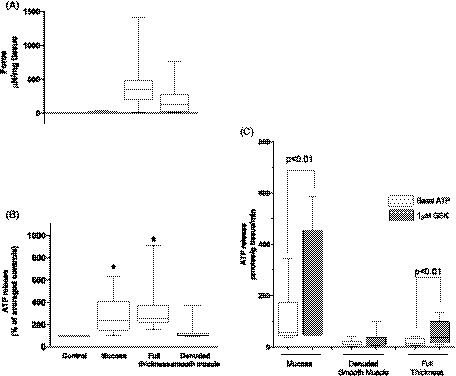
Effects of denuding GP bladder on GSK‐induced ATP release and contraction. Shown alongside mucosa and FT strips for comparison. A, Median force of tissue contractions generated by 1 μM GSK. No significant difference between FT and denuded smooth muscle strips. B, Corresponding median ATP release from same bladder strips. Denuded smooth muscle does not release ATP in response to GSK (n = 10). C, ATP release from same tissue strips expressed in ρM/g tissue/min. Significant ATP release from mucosa and FT, but not denuded tissue. GSK‐induced ATP release from mucosa was significantly greater than from FT (not shown). Basal ATP release from mucosa was significantly greater than basal ATP release from denuded and FT tissue (direct comparision plot not shown). Basal release from FT was not different to denuded. Median values [25%, 75%], mucosa and FT n = 8, denuded n = 10, *P ≤ .05, Wilcoxon's for drug intervention vs. control. Different data sets compared using Mann‐Whitney

To identify the tissue origin of ATP release in the bladder wall, the ATP release was examined in the smooth muscle and compared to those in mucosa and full‐thickness bladder strips. GSK evoked contractions in 70% of mucosa‐denuded smooth muscle strips with similar magnitude to FT strips, supporting that GSK‐induced contraction was mainly from the smooth muscle. However, the significant ATP release seen in the full‐thickness bladder tissue was lost, with only 16% of strips releasing ATP beyond basal levels (Figure 4A,B). Figure 4C shows the absolute quantity of ATP released from bladder strips in response to GSK (expressed as ρmoles/g tissue/min). Significant ATP release was evoked in both mucosa and FT tissue, but not in mucosa‐denuded smooth muscle. Mucosa released significantly more ATP per gram tissue than FT, implying the tissues underlying the mucosa in the FT strip are insignificant with regards to ATP release. Additionally, mucosa released significantly more *basal* ATP per unit tissue than both denuded smooth muscle and FT tissue (Figure 4C). This, and the fact that denuded smooth muscle is incapable of releasing significant amount of ATP in response to GSK, implies that most of, if not all GSK‐induced ATP release is from the mucosa and hence of urothelial origin.

### 3.4 Mechanisms underlying TRPV4‐induced mucosal ATP release

#### 3.4.1 Control experiment

**Figure 5:**
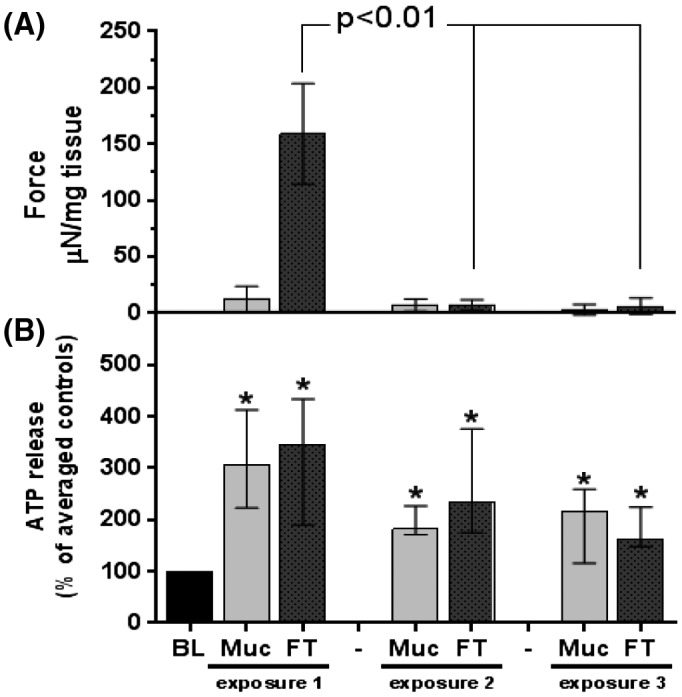
Effect of repeated exposure to GSK on GP bladder functional outputs. A, Force of contractions generated by three successive exposures to 1 μM GSK (1‐hour recovery period between each). No contractions observed in mucosa, a large contraction evoked in FT with first exposure, which is lost (significantly less) with successive exposures. B, Corresponding ATP release from same strips. All exposures significantly increase ATP release from basal levels (115 ± 27pmoles/g tissue/min). No significant difference in ATP release observed between successive exposures. Median values [25%, 75%], n = 10, *P < .05, Wilcoxon's

The majority of the experiments that follow required three repeated exposures of tissue to 1 μM GSK, with an hour recovery period between each and the second including an antagonist. The following control experiment assessed the reproducibility of ATP release in response to three successive exposures to GSK without antagonist. All three exposures significantly enhanced ATP release in both mucosal and FT bladder preparations, with no significant difference between any (Figure 5). A contraction was evoked with the first exposure, however the following exposures were incapable of eliciting further contractions suggesting possible desensitization of TRPV4 response on the smooth muscle and this was observed throughout all similar experiments in this study.

#### 3.4.2 Effect of TRPV4 blockade

**Figure 6:**
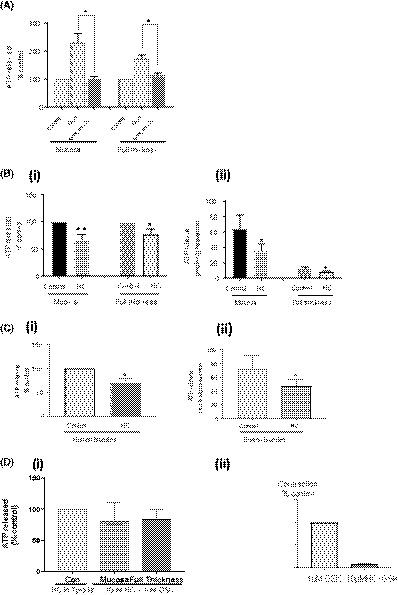
Effect of TRPV4 antagonism on ATP release. A, HC (5 μM) significantly reduced ATP release triggered by GSK (0.5 μM, 214 ± 82/pmoles/g tissue/min) in GP strips (n = 7; *P < .05). B(i), 1 μM HC significantly reduced basal ATP release in GP mucosal and FT strips, relative change (%). B(ii), Effect of HC on basal ATP release; data expressed as median release/g tissue/min. (n = 8; *P < .05, **P < .01); C(i), HC (1 µM) reduces basal ATP release in human mucosal biopsies (n = 7). C(ii), Effect of HC on basal ATP release in human mucosal samples; data expressed as % control and release/g tissue/min (n = 8; *P < .05). D, 10 µM HC also effectively inhibited 1 µM GSK‐induced ATP release (i) (n = 5) and smooth muscle contraction: (ii) (n = 4). Mean ± SEM; paired *t* test

5 μM HC‐067047 (HC), a selective TRPV4 antagonist, was chosen for predominant blockade (>90%) of TRPV4 receptors in the presence of 0.5µM GSK based on binding to the same receptor by two ligands with known affinities, while both agents were still within TRPV4‐receptor‐specific concentrations. The ATP release triggered by GSK (0.5 μM) was significantly reduced by HC to near basal levels in both tissue types (Figure 6A), demonstrating high specificity of GSK to TRPV4. A further test on 1 µM GSK with 10 µM HC also largely abolished the GSK‐induced ATP release as well as GSK‐induced muscle contractions, proving specificity of 1 µM GSK on urothelial ATP release and smooth muscle contractions (Figure 6D). HC (at a lower concentration in the absence of GSK, 1 µM) also significantly reduced basal ATP release from mucosa (Figure 6B). Basal ATP release was significantly reduced after 10 minutes exposure. These data suggest that TRPV4 channels are open in the intrinsic environment of bladder tissues, especially in the urothelium and hence the functional importance of this receptor in controlling ATP release from the bladder tissues.

#### 3.4.3 Specific pathway for TRPV4‐induced ATP release

**Figure 7:**
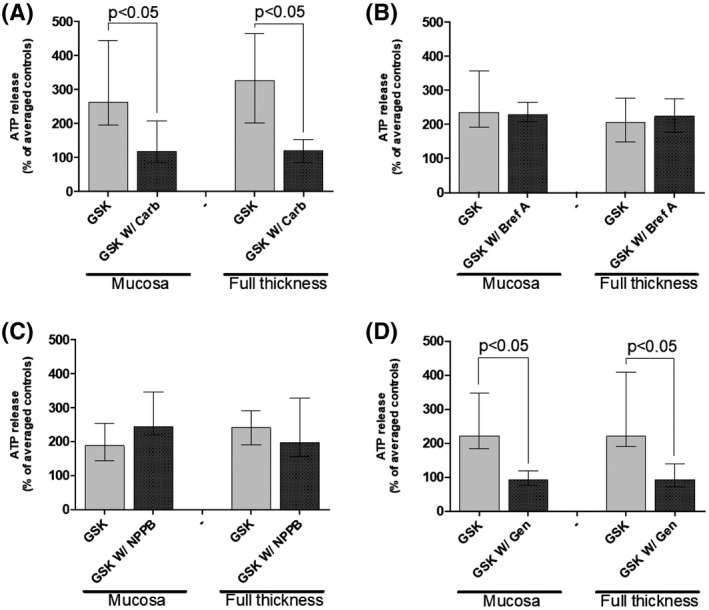
Effect of various inhibitors on GSK‐induced ATP release from GP bladder strips. A, Carbenoxolone (100 μM; connexin/pannexin channel blocker) significantly reduces GSK‐induced (n = 9). B, Brefeldin A (10 μM; vesicular transport inhibitor) has no effect on GSK‐induced ATP release. C, NPPB (100 μM; inhibits Ca^2+^‐sensitive chloride currents) has no effect on GSK‐induced ATP release (n = 6). D: Genistein (100 μM; selective protein tyrosine kinase inhibitor) significantly reduces GSK‐stimulated ATP release (n = 8). Median [25%, 75%], Wilcoxon's. Control: 282 ± 72 pmoles/g tissue/min

The two fundamental pathways for ATP release from the cell cytoplasm to extracellular space (conductive and exocytotic^52^, ^53^) were further investigated for TRPV4‐induced mucosal ATP release. The pannexin/connexin channel inhibitor carbenoxolone (100 μM) significantly reduced GSK‐induced ATP release in both tissue types by approximately 90% (Figure 7A). Brefeldin A (10 μM), an inhibitor of vesicular transport‐mediated ATP release, had no effect on GSK‐induced (TRPV4‐activted) ATP release in either tissue type (Figure 7B). These findings suggest that the observed ATP is released via conductive pathways, with little or no involvement of vesicular transport.

Various other channels and signaling molecules were investigated to further elucidate the pathway underlying TRPV4‐mediated ATP release. Limited previous literature suggests that ATP release through hemichannels is associated with activation of Cl^−^ channels.^54^ Therefore, the effect of NPPB (Ca‐activated Cl channel blocker, 100 μM) was investigated; however, this was ineffective in reducing GSK‐induced ATP release (Figure 7C). Genistein (selective protein tyrosine kinase inhibitor, 100 μM), however, suppressed GSK‐induced ATP release to below basal levels in both tissue preparations (Figure 7D), suggesting a role for tyrosine protein kinases in either the downstream signaling pathway for TRPV4‐mediated ATP release or in the modulation of the activity of TRPV4 channels themselves.

#### 3.4.4 Role of Ca^2+^ in TRPV4‐mediated ATP release

**Figure 8:**
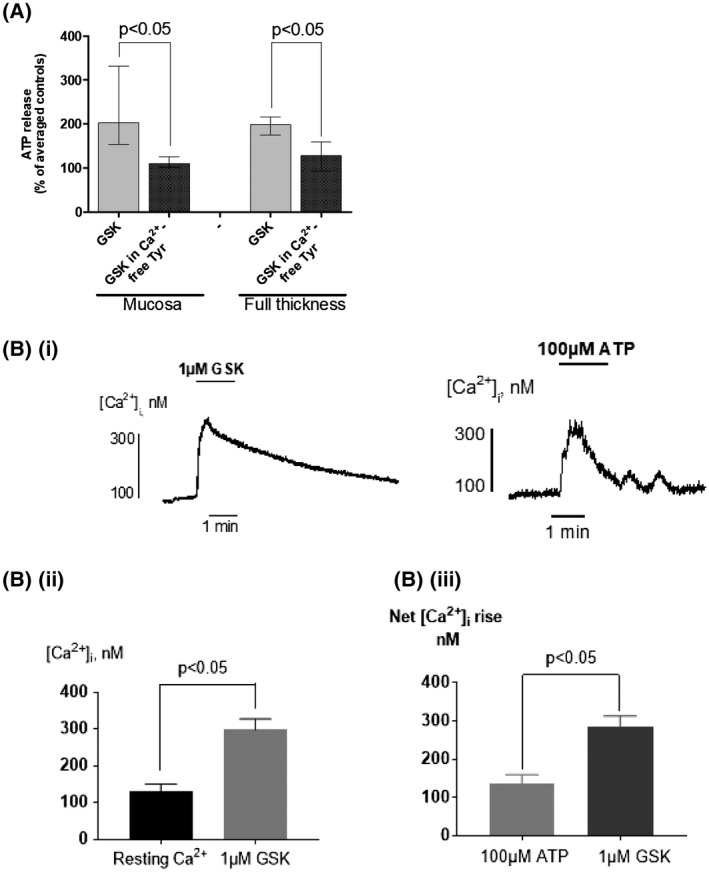
Role of Ca^2+^ in GSK‐induced ATP release from GP bladder tissue. A, Removal of Ca^2+^ from Tyrode's superfusate significantly reduces GSK‐stimulated (1 μM) ATP release (Median, [25%, 75%], n = 8, Mann‐Whitney)); control: 166 ± 55 pmoles/g tissue/min. B(i), GSK (1 μM) generates a Ca^2+^ transient (left) in a freshly isolated single urothelial cell from GP bladder; ATP‐induced Ca^2+^ transient (right). B(ii), Averaged data; GSK (1 μM) evokes a significant rise in [Ca^2+^]_i_, (mean ± SEM, n = 7, *P* < .05). B(iii), Averaged data; GSK (1 µM) induced [Ca^2+^]_i_ rise (n = 9) vs ATP (100 µM) induced[Ca^2+^]_I_, (n = 14); *P* < .05, Mean ± SEM, unpaired *t* test

The Ca^2+^ dependence of TRPV4‐mediated ATP release was explored by removal Ca^2+^ in a Ca^2+^‐free extracellular environment. The stimulating effect of 1 μM GSK on ATP release was abolished in the absence of extracellular Ca^2+^ (mucosa: 90% reduction, FT: 70% reduction; Figure 8A; Ca^2+^‐free modified Tyrode's solution used is described in *Solutions*). This suggests a key role of Ca^2+^ in mediating ATP release from the urothelium. The role of Ca^2+^ in TRPV4 activation was further examined in freshly isolated urothelial cells. Live‐cell Ca^2+^ imaging in single urothelial cells demonstrated a large rise in intracellular Ca^2+^ in response to TRPV4 activation (Figure 8B(i)). The averaged data from seven bladders revealed that TRPV4 activation (GSK, 1 μM) significantly raised [Ca^2+^]_i_ from resting levels, providing further evidence for the involvement of Ca^2+^ in TRPV4‐mediated signaling (Figure 8B(ii)). Importantly, the magnitude of TRPV4 activation induced Ca^2+^ rise is much greater than that induced by the major physiological activator ATP in the same cells (Figure 8B(iii)). This demonstrates that Ca^2+^ response is a key signaling pathway for TRPV4 activation and also the functional importance of TRPV4 receptors in urothelial function. Furthermore, the Ca^2+^ rise by TRPV4 activation has a long duration and is sustained after removal of the agonist, which reveals a long open state of the channel (Figure 8B(i)). Thus, these data support that extracellular Ca^2+^ entry, resulting in increased intracellular Ca^2+^, is involved in TRPV4‐mediated ATP release.

#### 3.4.5 Effect of TRPV4 inhibition on stretch‐evoked ATP release

**Figure 9:**
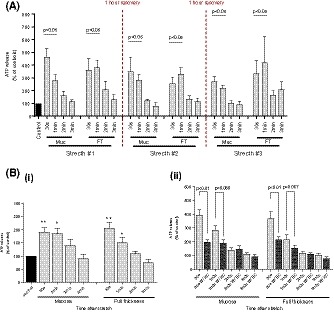
Effect of TRPV4 inhibition on mechanical stretch‐induced ATP release from GP bladder tissues. A, Control experiment (n = 5). Mechanical stretch evoked significant ATP release at 30 seconds and 1 minute during each repeated stretch. The ATP release subsided after 2 minutes. No significant difference was observed in 30‐second ATP release and 1‐minute ATP release between the stretches. B, Stretch‐induced ATP release during inhibition of TRPV4 receptors with HC‐067047 (second stretch during protocol). B(i), Mechanical stretch stimulated significant ATP release (n = 11); control: 111 ± 28 pmoles/g tissue/min. B(ii), Stretch‐induced ATP release with (dark grey bars) or without (light grey bars) inhibition of TRPV4 (HC‐067047; 1 μM). TRPV4 inhibition significantly attenuates stretch‐induced ATP 30 seconds after mechanical stimulation (n = 11, Light grey bars: Averaged ATP release from pre‐ and post‐stretch interventions. Dark grey bars: ATP release from middle stretch intervention with 1 μM HC‐067047). Mean ± SEM, *P < .05, **P < .01

The question as to what extent TRPV4 is involved in stretch‐induced ATP release in intact bladder tissue was also examined. Mucosal and FT strips (5‐6 mm) were stretched to 150% of their original length for 30 seconds using a micromanipulator, and ATP release was measured during and after stretch (parameters based on preliminary work). Strips were successively stretched three times, with 1‐hour recovery periods. A control experiment assessed the viability and reproducibility of this protocol (Figure 9A). 150% stretch evoked significant release of ATP at 30 seconds and 1 minute after initial stretching, which subsided after 2 minutes. These dynamics of ATP release were reproducible for a second and third stretch. The increased ATP release at 30 seconds and 1 minute in both tissue preparations during all three stretches have a *P* value < 0.05 compared to controls for n = 5 and there was no statistical difference in stretch‐induced ATP release from the tissues between different runs of sequential stretches. This information was sufficient to perform experiments using antagonists to assess the role of TRPV4 in stretch‐induced ATP release.

TRPV4 was antagonized using HC (1 μM) during the second stretch. Tissue stretch during inhibition of TRPV4 with HC still raised ATP release above basal levels in both tissue preparations at 30 seconds and 1 minute (Figure 9B(i)). However, the stretch‐evoked ATP release during TRPV4 inhibition was significantly less than that evoked without inhibition at 30 seconds in both tissue preparations and at 1 minute also in FT (Figure 9B(ii)). These values translate to approximately a 50% reduction in the stretch‐evoked ATP release when inhibiting TRPV4 (mucosa; 30 seconds—61% reduction, 1 minute—46% reduction, FT; 30 seconds—49% reduction, 1 minute—70% reduction). This suggests that TRPV4 is partly but not exclusively responsible for the stretch‐induced ATP release from the bladder mucosa.

### 3.5 Effect of TRPV4 activation on bladder reactive oxygen species (ROS) production

**Figure 10:**
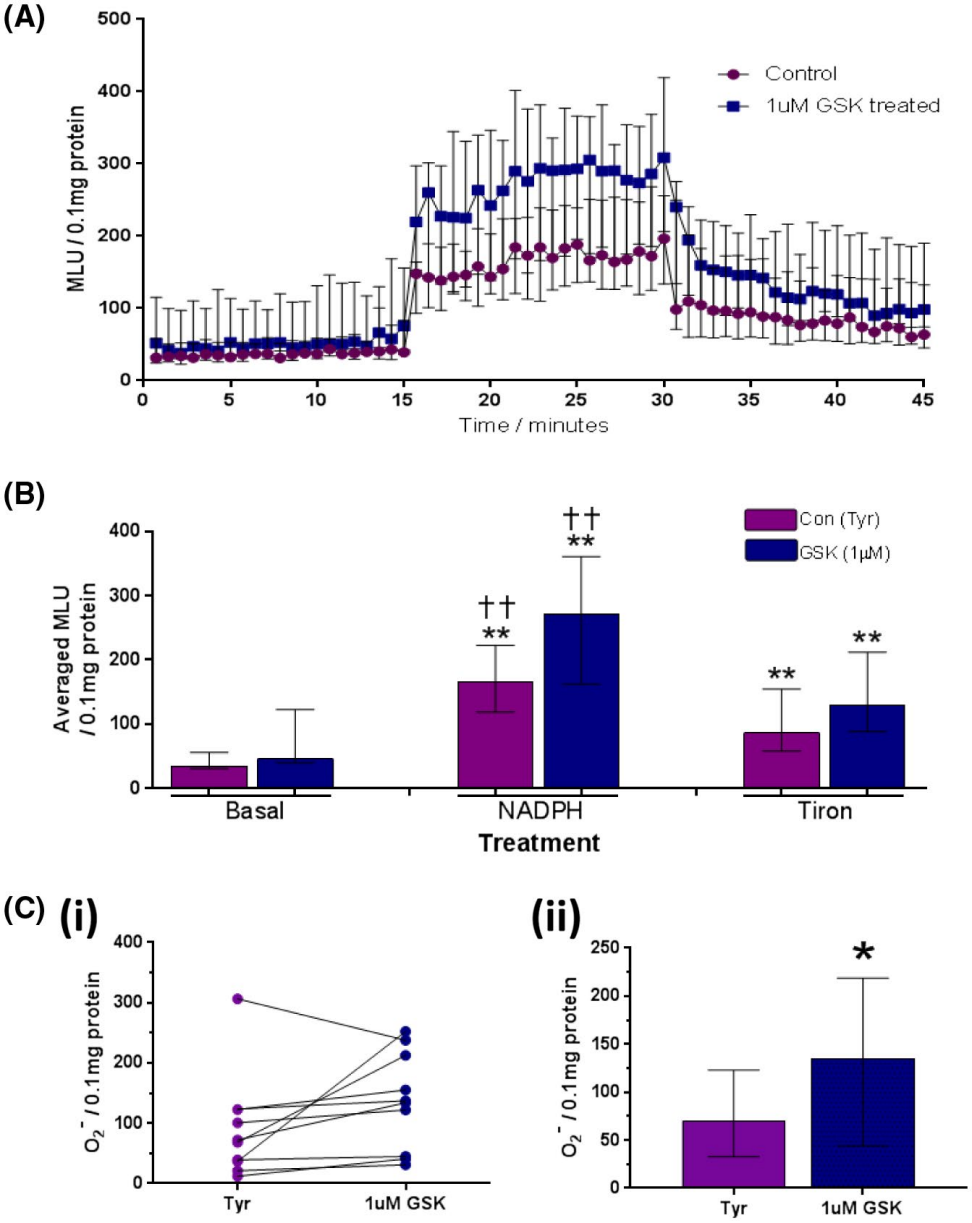
Effect of 1 μM GSK on ROS and O_2_^−^ production in homogenized GP bladder tissue. A, Kinetics of ROS production over experimental time‐course. 0‐15 minutes; basal ROS production, 15‐30 minutes; ROS production after 100 μM NADPH, 30‐45 minutes; ROS production after 5 mM tiron (O_2_^−^ scavenger; n = 10). B, Median light units (MLU) averaged across 15‐minute period of each treatment. No significant difference observed between groups during any treatment. NADPH (100 μM) significantly enhanced ROS from basal levels in both tissues (**). ROS production in the presence of Tiron was significantly less than during NADPH treatment (††), but still significantly greater than basal levels (**; n = 10). C, Tiron‐sensitive (inhibited) component (O_2_^−^‐specific production): (i): Paired dot plot‐‐difference in O_2_^−^ production between control and GSK‐treated FT strips from each bladder investigated (n = 10). (ii): Averaged O_2_^−^ production. Pre‐treatment with 1 μM GSK (TRPV4 activation) stimulates significantly greater O_2_^−^ production than controls (n = 10). Median values [25%, 75%], *P ≤ .05, **P ≤ .01, Wilcoxon's

Inflammation and oxidative stress are closely related, and both participate in aging and pathogenesis of many chronic conditions. The ability of TRPV4 channel to generate ROS was thus investigated. Superoxide (O_2_^−^) production was measured using lucigenin chemiluminescence in homogenized GP FT strips after pre‐treatment with either control Tyrode's solution or 1 μM GSK. In the absence of added agents, basal luminescence intensity in FT tissue lysates was minimal and did not differ between control and treated groups (MLU/0.1 mg protein: Tyrode's con—35 [30, 55], GSK—46 [39, 122]; Figure 10B). Addition of NADPH (100 μM) significantly increased the luminescence intensities from basal levels in both groups, representing NADPH‐dependent ROS production. After addition of the O_2_^−^ scavenger Tiron (10 mM), NADPH‐dependent ROS production was significantly suppressed, suggesting most of NADPH‐dependent ROS production is superoxide. Addition of Tiron enabled the proportion of ROS composed of O_2_^−^ to be determined. This revealed that activation of TRPV4 receptors by pre‐treatment with GSK significantly enhances O_2_^−^ production in bladder tissue above that of control tissue (MLU: Tyrode's control—70 [33, 123], GSK—136 [44, 219]; Figure 10C(ii)). TRPV4‐induced O_2_^−^ production is a novel mode of action which may affect bladder signaling and alter oxidative stress levels with further downstream implications.

### 3.6 Effect of aging on TRPV4 expression and function

Recent evidence suggests that aging affects urothelial function. The effect of aging on TRPV4 expression and function was thus examined.

#### 3.6.1 TRPV4 protein expression

**Figure 11:**
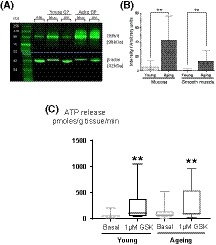
Effect of aging on TRPV4 expression and function in GP bladder tissue. A, Representative blot of TRPV4 (98 kDa, probed using *Abcam* primary Ab) and β‐actin loading control (42 kDa; RBL: rat brain lysate Muc: mucosa, SM: smooth muscle). B, Quantified data of TRPV4 expression by densitometry. Each TRPV4 band is normalized to corresponding loading control. Samples duplicated and averaged. Aging tissues have significantly higher TRPV4 expression than young (n = 5). C, GSK significantly enhanced ATP release from mucosal strips in both age groups. The quantity of basal and GSK‐induced ATP release per gram tissue was not significantly different between age groups (young GP n = 8, aging GP n = 14). Median values [25%, 75%], **P ≤ .01; Wilcoxon for GSK vs basal; Mann‐Whitney for Aging vs Young

The representative blot in Figure 11A shows clear bands at the same molecular weight (98 kDa) as the positive control, proving presence of TRPV4. Visual inspection of the band intensity and size shows clearly that the expression of TRPV4 is greater in aging tissue than in the young. Quantification by densitometry further revealed a significantly greater median TRPV4 expression (eightfold) in aging mucosa than young (arbitrary units normalized to loading control aging: 42.9 [25.7, 75.6], young; 5.8 [2.1, 14.3]) and (16‐fold) in smooth muscle (aging; 13.5 [10.1, 28.3], young; 0.8 [0.6, 3.4]; Figure 11B). These data suggest that TPPV4 expression is up‐regulated during aging.

#### 3.6.2 TRPV4‐induced contractility and ATP release

GSK was able to trigger ATP release from bladder mucosa in aging bladders. There was no significant difference in the quantities of ATP released from the mucosa per gram of tissue in response to TRPV4 activation between age groups (ρmoles/g tissue/min: Young, con; 47 [38, 63], GSK; 105 [45, 379]; Aging, con; 72 [37, 134], GSK; 96 [73, 543]; Figure11C). Similarly, contractions evoked by GSK in young and aging FT strips were not significantly different (not shown).

### 3.7 Relevance to human conditions

To show the relevance of the above findings from animal tissues to human conditions, evidence for the expression of TRPV4 proteins and its functional role were examined in freshly obtained human bladder mucosal biopsies.

#### 3.7.1 Evidence of TRPV4 expression in human bladder tissues

Immunofluorescence staining shows positive staining for TRPV4 antibody. This was observed in the four human biopsies assessed (Figure 1A). The positive immunoreactivity was observed across all layers of the bladder wall. TRPV4 immunoreactivity was greater in urothelium than suburothelium and DSM for each of the four biopsies examined, similar to data obtained from GP bladder tissues. These data show that TRPV4 proteins are expressed in the bladder wall, and the urothelium has the highest level of expression in human bladders.

Western blot measurement was further performed in human tissue. TRPV4 expression was detected in three of the four human mucosal biopsies examined (non‐OAB cohort). The same RBL positive control sample used for GP blots was used here, providing reassurance of correct band size. Once again, clear bands were observed at the same molecular weight (~98 kDa) as the positive control for three of the four mucosal lysates assessed, thereby confirming presence of TRPV4 (Figure 2D) in human bladder mucosa.

#### 3.7.2 The functional role of TRPV4 receptor and its regulation in human conditions

Further to the demonstration of TRPV4 expression in human bladder tissues, the function of TRPV4 receptor was examined.

##### Effect of TRPV4 activation on ATP release and contractions in human mucosal biopsies

The two key physiological outputs for bladder mucosa and smooth muscle, ATP release and muscle contractility, respectively, were investigated when TRPV4 receptor was selectively stimulated with the specific activator GSK1016790A.

**Figure 12:**
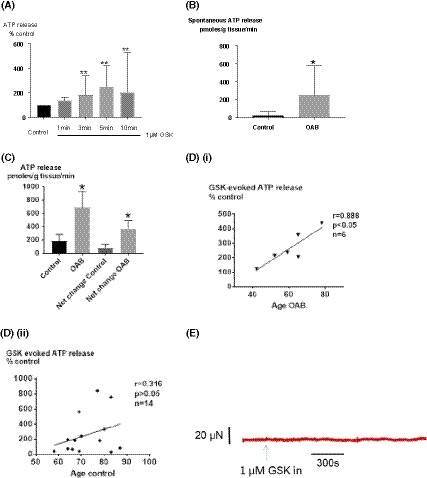
Effect of TRPV4 activation on human bladder biopsies with or without OAB symptoms. A, GSK (1 μM) significantly enhanced ATP release from human mucosal biopsies (non‐OAB control). B, Higher spontaneous ATP release from mucosal biopsies in OAB patients (n = 6) (vs. non‐OAB control, n = 14, *P* < .05); C, Greater GSK‐induced ATP release per unit mass of mucosa in OAB group and net increase in ATP release in OAB group compared with controls (*P* < .05); D correlation between mucosal ATP release and age D(i), Close correlation in OAB samples; D(ii), No correlation in control patients. E, Example trace of tension during challenge with 1 μM GSK in human mucosal biopsies: little contraction was produced by GSK application. Median, [25%, 75%], Mann‐Whitney

GSK (1 μM) significantly enhanced ATP release, with an onset 3 minutes after initial exposure, with the peak at 10 minutes in non‐OAB biopsies (n = 10) (Figure 12A). This response is slightly delayed compared to GP tissue, which evokes ATP release after 1 minute. However, the overall positive response was similar to GP tissue.

Mucosal sheets did not produce contractions in response to GSK (Figure 12E). These data show TRPV4 activation does not cause mucosal contractions in human tissue, consistent with findings in GP bladders.

##### Effect of TRPV4 inhibition on basal ATP release

TRPV4 inhibition (HC, 1µM) in non‐OAB human mucosa significantly reduced basal ATP release. This demonstrates that there is intrinsic activity of TRPV4 receptors in human bladder mucosa. The data show species similarity to GP and support functional importance of this receptor in maintaining basic physiological function of the bladder across the species (Figure 6C).

##### TRPV4 activity in pathological bladders

The ability of the urothelial tissue to release ATP was assessed in human OAB bladder mucosa. Bladder mucosal biopsies from six patients with OAB were examined. Spontaneous basal release of ATP from bladder mucosa was increased in OAB bladders compared to that in non‐OAB bladders (ρmolar ATP release: OAB, 250 [70, 575]; control: 23 [19, 64], *P* < .05, Figure 12B). GSK enhanced ATP release from basal levels in all OAB samples. The relative increase in ATP release from basal levels was similar in both OAB samples and non‐OAB controls (OAB: 263 ± 46% of basal level *vs*. control: 258 ± 72% (n = 14); *P* > .05), suggesting that the proportionate drug effect of GSK on ATP release is similar in both groups. However, importantly, the quantity of ATP released by TRPV4 agonist in OAB samples was greater than that in control bladders; the peak ATP level and the net increase in ATP release were much higher from the overactive bladders (peak ATP levels, ρmoles/g tissue/min; OAB, 693 ± 239 (n = 6) *vs*. control, 190 ± 98 (n = 14), *P* < .05; net increase: OAB, 190 ± 98 (n = 6) *vs.* control, 88 ± 49 (n = 14), *P* < .05, Figure 12C). The proportional increase in ATP release triggered by GSK was not correlated with age in the non‐OAB control group (Figure 12D(ii)). However, this increase was positively correlated with age in the OAB group (Figure D(i)) These data suggest that urothelial tissue in human OAB bladders spontaneously releases more ATP than that in control bladders and greater quantity of ATP is released from the OAB urothelium by TRPV4 activation. Importantly, the fractional increase in ATP release from OAB samples in response to GSK is closely correlated to age, suggesting a specific role of aging in regulation ATP release in OAB development.

## 4 DISCUSSION

The data from this study show that TRPV4 receptors are differentially expressed throughout the bladder wall with predominant expression in the urothelium, and that TRPV4 receptors regulate key physiological endpoints in both the urothelium and bladder smooth muscle, emphasizing the functional importance of this receptor in the bladder. Of significance, specific TRPV4 activation evokes significant ATP release from native bladder tissue and constitutive TRPV4 activity leads to ATP release, with the urothelium identified as the source for such ATP release. A Ca^2+^‐ and protein tyrosine kinase‐dependent signal transduction pathway via pannexin channels underlying TRPV4‐induced ATP release has been elucidated, a novel action of TRPV4 to produce superoxide identified, and pathological significance and human relevance demonstrated. To our knowledge, this is the first study to use native animal and human bladder tissue to reveal the mode of action of TRPV4 receptors in native bladder tissue, either stretch‐independent or stretch‐related, and to demonstrate pathological and human relevance. These findings suggest species similarity and identify an age‐dependent change in expression and increased ATP release in overactive bladders. These have important implications in bladder pathophysiology.

### 4.1 Expression of TRPV4 receptor in bladder

Most of early studies on TRPV4 expression by immunostaining or Western blot mainly focused on one tissue compartment of the bladder with a single method; thus, relative tissue distribution has not been determined and cross‐species comparison is unknown.^35^, ^36^, ^37^ The current study is a conclusive demonstration of the expression and localization of TRPV4 throughout guinea‐pig and human bladder tissues. In this study, TRPV4 expression was shown by immunostaining and Western blotting in both human mucosa and guinea‐pig bladder tissues with detailed information on its tissue distribution. Confocal immunofluorescence images demonstrated TRPV4 expression in the urothelium, suburothelium and detrusor smooth muscle of guinea‐pig bladder tissue, with highest expression in the urothelium. Western blotting results provide further evidence for TRPV4 expression in both mucosa and detrusor smooth muscle, with significantly higher expression in the mucosa. Together, these data identify the urothelium as the predominant site of TRPV4 expression in the bladder. To our knowledge, this is the first study to show expression of TRPV4 in guinea‐pig bladder using these two complementary techniques, and to indicate the tissue localization. Importantly, TRPV4 expression was also demonstrated in human mucosal biopsies which corroborates previous preliminary observations.^37^ Furthermore, we identified TRPV4 receptors in both guinea‐pig and human suburothelial cells which has not been shown before. Because suburothelial cells (interstitial cells/myofibroblasts) are in series with urothelial cells to work as a functional syncytium to amplify urothelial signals,^10^ this finding has physiological importance. Of pathological significance, we demonstrated an increased TRPV4 expression in both mucosa and detrusor smooth muscle from aging bladders. This may have implications on TRPV4 function and bladder physiology during the aging process.

### 4.2 The basic mode of action and functional role of TRPV4 receptor in bladder

The role of TRPV4 in normal bladder function has been previously investigated by several studies.^35^, ^38^, ^51^, ^55^ These earlier studies have provided important basic biological actions of TRPV4 receptors and a physiological role in bladder function. In particular, the Nilius group^35^ has established that global TRPV4 knockout mice exhibit reduced frequency of voiding contractions in cystometry and suppressed spontaneous contractions in bladder strips; a decreased intravesical stretch‐evoked ATP release was found in isolated whole bladders. However, measurement of ATP release directly from isolated urothelium/mucosa was not performed in this study and tissue‐specific actions remain to be established. Other previous in vitro studies focused mainly on cultured urothelial cells and used stretch as a method to activate TRPV4 which may trigger many other non‐specific stretch‐sensitive processes. However, the mode of action of TRPV4 in native urothelial tissue layer, in particular, by specific activation of this receptor, independent of stretch, has not been examined and the mechanisms of action are poorly understood. To accentuate the physiological relevance, we assessed the role of TRPV4 by selective activation of this receptor by GSK1016790A without the cofounding influence of stretch‐induced non‐specific effects. In the present study, we examined the role of TRPV4 on the ability of the urothelium to release ATP which acts as a novel sensory mediator to activate suburothelial sensory nerves and other purinoreceptor expressing interstitial cells and the contractility of detrusor smooth muscle which governs the voiding function of the bladder. The results provide multiple lines of evidence to establish a functional role for TRPV4 in urothelial ATP release. Selective activation of TRPV4 using GSK1016790A evoked significant and reproducible release of ATP from the mucosa of human and guinea‐pig bladders, demonstrating common functionality of TRPV4 between species. As such, this serves as evidence that the TRPV4 activity observed in these animal models can be translated to humans with confidence. Importantly, mucosal strips released far greater quantities of ATP per unit of tissue mass than full‐thickness strips, suggesting the majority of ATP released by the bladder in response to TRPV4 activation originates from the urothelium. Removal of the mucosa resulted in near complete loss of GSK/TRPV4‐induced ATP release, further supporting the urothelium as the source of ATP release. Furthermore, there is no correlation between the quantity of ATP release and magnitude of contraction, implying the two phenomena are relatively independent.

Antagonism of TRPV4 with HC‐067047 completely abolished any GSK‐induced ATP release, confirming selectivity of GSK. This is in contrast with previous studies, where stretch or non‐selective 4α‐PDD was used for TRPV4 activation and the multi‐action inhibitor ruthenium red was used as a TRPV4 inhibitor and thus the specific properties of TRPV4 could not be determined. Importantly, we show that specific activation of TRPV4 receptor by agonist without stretch can release significant amount of ATP. This implies that activation of the TRPV4 receptor can occur by chemical signals encountered in the tissue environment and in particular under inflammatory conditions with release of endogenous inflammatory mediators, even when the bladder is relaxed without any mechanical stimulus from bladder distension or contractile activity. Indeed, burning sensations occur in empty bladders during bladder infection and non‐infectious inflammatory cystitis. This suggests that TRPV4 receptors can sense noxious chemical signals and thus serve as a sensor of pathology. Furthermore, antagonism with HC‐067047 reduced basal ATP release in the absence of GSK in both GP and human mucosa, which has not been reported before. This implies that TRPV4 is active without exogenous activators and is therefore activated by endogenous ligands in the tissue that maintains basal ATP release by sustaining a “TRPV4 tone.” The native mechano‐ and osmotic environment of bladder tissue may serve as low strength mechanical signal. More importantly, the endogenous chemical environment may also provide a constant chemical stimulus. Changes to these conditions and the temperature increase during inflammation may aggravate noxious stimuli to up‐regulate TRPV4 activity. All these emphasize important pathological impact. Currently, epoxyeicosatrienoic acids (EETs) are the only known endogenous activators of TRPV4 identified.^32^, ^33^ TRPV4 channel activity can also be enhanced by the Ca^2+^‐CaM complex and IP_3_.^56^, ^57^ Both spontaneous intracellular Ca^2+^ release and IP_3_ pathways associated with P2Y receptors exist in urothelial cells^58^ which may enhance TRPV4 activity. Src‐family tyrosine kinases (SFKs) can also sensitize TRPV4 channel activity and are thought to mediate the channel's responses to mechanical/hypotonic stimuli.^59^, ^60^ SFKs are ubiquitously expressed including urothelial cells^61^ and regulate a vast number of cellular processes and responses to oxidative stress (reviewed elsewhere^62^, ^63^) and may be available for basal TRPV4 activation, possibly sensitizing the receptor for lower level stimulation. How other endogenous chemical mediators affect TRP4 activity remains to be explored. A role for urothelial TRPV4 in spontaneous bladder contractility has been previously demonstrated.^35^ Removal of the urothelium and knockout of TRPV4 resulted in reduced spontaneous activity, suggesting that urothelial TRPV4 is responsible for this spontaneous activity, and not DSM TRPV4. Our identification of significant ATP release from the native mucosa suggests a role for TRPV4 derived basal ATP release from the urothelium in driving this TRPV4‐dependent spontaneous activity observed previously.

A role for TRPV4 expressed in the DSM has been identified both here and in previous studies.^55^, ^64^, ^65^, ^66^ TRPV4 activation evoked sustained contractions in intact bladder strips of similar magnitude to those evoked by the cholinergic agonist carbachol. These contractions were also observed in denuded DSM strips and therefore suggest that direct activation of TRPV4 receptors in the DSM can cause DSM contractions, although additional small contractions could result from an indirect effect of ATP release. Consistent with findings from previous studies, this study identifies the presence of functional TRPV4 receptors in the DSM and further reveals the ability of TRPV4 to directly contract bladder muscle with a force equaling that of muscarinic receptor activation. The magnitude of TRPV4 activation‐induced smooth muscle contraction adds weight to its physiological relevance. Thus, these findings reveal that TRPV4 activity in DSM may indeed influence bladder pathologies associated with muscle over‐activity or underactivity.^67^, ^68^

The demonstration of TRRV4‐mediated ATP release in native bladder tissue suggests that TRPV4 can influence the physiology of the bladder. It is currently thought that the urothelium senses distension in the bladder during filling and communicates this to the underlying tissues by release of ATP as a chemical signal. This may be a fundamental process in the bladder's overall ability to sense fullness, whereby the extent of bladder distension (equating to urine volume) is relayed to underlying afferent nerves via an ATP signal generated, in part, by TRPV4. ATP acts on P2X3 and P2X2/3 receptors on suburothelial sensory nerves.^3^, ^5^, ^69^ These nerves continuously convey information regarding urine volume to the PMC for voluntary bladder control and perception of bladder fullness, initiating urges to void at appropriate volumes that induce pressures of 20‐25 mm Hg permitting correct periodic storage and voiding of urine. Pain is perceived once certain harmful volumes producing pressures of 30‐50 mm Hg are reached.^70^

### 4.3 Stretch and TRPV4 receptors

Mechanical stretch evokes a reproducible ATP release from the mucosa. TRPV4 blockade results in a 50% reduction of this ATP release. This finding provides new evidence for a specific role of TRPV4 in stretch‐induced ATP release from the native urothelium and its quantitative contribution. This is different from the previous report on cultured urothelial cells where ATP release from these cultured cells by stretching the cell adhered to silicone was completely abolished by ruthenium red, implying 100% contribution of TRPV4 channel to stretch‐induced ATP release under these conditions.^38^ This difference can be explained by the non‐specific inhibitor used previously and the different stretch method (silicone gel deformation) which is different from mechanical bladder distension. More importantly, the cultured cells may behave differently from native cells and tissue and the results cannot be directly extrapolated to native cells and tissue. It is unlikely that all stretch‐induced cell responses are solely mediated by TRPV4 channels. ENaC channel, gadolinium‐sensitive stretch‐activated channel, and Piezo1 channel have been shown to contribute to stretch‐induced ATP release in the urothelium.^9^, ^21^, ^71^ Our findings suggest a physiological role of TRPV4 channels in regulating urothelial ATP release in the normal micturition cycle in response to a physiological stimulus—bladder distension/stretch. The mechanisms by which stretch activates TRPV4 channels have been studied previously in other tissue types and it is currently thought that the process requires activation of TRPV4 by EETs, generated by PLA_2_‐dependent pathway,^32^, ^72^ or phosphorylation by SFKs,^59^, ^60^ or indeed both. However, the possibility of direct activation by mechanical force has not been excluded^73^ and therefore further research is required to fully establish the mechanisms underlying stretch activation of TRPV4. The downstream mechanisms underlying TRPV4‐induced ATP release are elucidated in the present study. We show that protein tyrosine kinase activity is required for GSK‐induced ATP release. This was not observed in a previous study on cultured cells using the TRPV4 agonist 4α‐Phorbol 12‐13‐dicaprinate (4αPDD), however that was likely due to different activation mechanisms.^74^ Furthermore, the signaling pathways in cultured cells may be different from those in native cells emphasizing the need to examine these mechanisms in native tissue.

### 4.4 Mechanisms of action

This study found that GSK/TRPV4‐induced ATP release is mediated mainly by conductive pathways (connexin and pannexin channels; 90%), with little or no contribution from vesicular transport. Pennexin‐conductive fluxes have been shown to contribute to intrinsic ATP release from the urothelium by our earlier study.^24^ The current study shows that this pathway is mainly involved in TRPV4‐mediated ATP release. The process was also tightly dependent on extracellular Ca^2+^ entry, the main action mediated by TRPV4 channels. This was further evidenced by live‐cell calcium imaging, which identified a rise in [Ca^2+^]_i_ upon TRPV4 activation in freshly isolated GP urothelial cells. The far greater response of Ca^2+^ rise by TRPV4 activation than that by activation of the main functional receptor‐purinergic receptor in the urothelium by ATP shows crucial role of TRPV4 receptors in urothelial function and intracellular Ca^2+^ rise as the key second messenger in TRPV4 signaling. Blockers of Ca^2+^‐activated chloride channels had no effect on GSK‐induced ATP release, excluding them from the mechanism. However, inhibition of protein tyrosine kinase significantly suppressed ATP release, suggesting that protein tyrosine kinase, which is associated with cytoskeleton restructuring and focal adhesion proteins,^75^ and other kinase‐dependent cellular pathways is one of the downstream mechanisms. Previous studies on cultured cells showed that stretch or the non‐specific activator 4aPDD could release ATP or induce a Ca^2+^ rise, suggesting the potential role of Ca^2+^ rise in ATP release; however, the causal relationship was not established, and the upstream and downstream coupling molecules were not examined. The collective findings from the present study suggest a mechanism whereby stretch or non‐stretch chemical activators activate TRPV4 receptors via SFK‐mediated phosphorylation of the channel; TRPV4 mediates Ca^2+^ influx raising [Ca^2+^]_i_; which activates a Ca^2+^‐dependent, hemichannel‐mediated ATP release mechanism, likely through pannexin‐1 channels^76^ and with intracellular tyrosine kinase as downstream pathway.

The study also discovered a long‐open state of the TRPV4 channels as evidenced by a prolonged intracellular Ca^2+^ rise in urothelial cells and sustained contractions in smooth muscle not observed with purinergic or muscarinic activation. This suggests that TRPV4 receptors can maintain activity in response to constant low levels of noxious stimuli during inflammation and ischemic conditions such as outflow obstruction and bladder hypertrophy. This is consistent with basal activity as demonstrated by the action of the TRPV4 antagonist in the absence of exogenous stimuli and has important pathological implications.

A fundamental role for TRPV4 in urothelial transduction of sensory information regarding bladder distension has been identified by this investigation, which may also have implications in bladder overactivity and pain. Urothelial TRPV4 may not only mediate volume‐dependent pain perception in the bladder under physiological conditions but may also induce pain and respond to inflammatory stimuli as a result of various pathologies including bladder inflammation. Changes in the expression and function of TRPV4 that result from aging or pathology that have indeed been identified, may influence painful bladder. TRPV4‐mediated nociception in the rat has been shown to be enhanced by PGE_2_, a hyperalgesic inflammatory mediator.^74^ As such, inflamed bladders could enhance TRPV4 mediated nociception in the same manner, implicating TRPV4 in painful bladder pathologies.

### 4.5 ROS production: a novel mode of action for TRPV4

The current study identified a further, potentially pathologic output of TRPV4, where TRPV4 activation appears to increase bladder superoxide (O_2_^−^) production. O_2_^−^ plays physiological roles in cellular signaling, for example in cell growth, where low levels stimulate cell proliferation^77^ and excessive O_2_^−^ contributes to oxidative damage and many pathological conditions. TRPV4 activation can initiate mitochondrial ROS production in human coronary artery strips for vasodilation.^78^ Our novel findings suggest that TRPV4 activation, possibly by stretch, induces NADPH‐dependent ROS production for ROS‐mediated physiological processes in the urothelium. More importantly, TRPV4‐mediated ROS production may contribute to oxidative stress and bladder pathologies.

A previous study shows that intravesical administration of hydrogen peroxide induces overactivity of the bladder.^79^, ^80^ Substance P has been reported to cause bladder smooth muscle contractions and also induce ROS production.^79^, ^80^ In addition, ROS significantly up‐regulates the phosphorylation of TRPV4 by SFKs,^60^ presenting a positive feedback on TRPV4, where TRPV4‐mediated ROS production enhances receptor activity. As ROS overproduction is an important pathological process in inflammation, many chronic diseases and aging; the ability of TRPV4 to stimulate ROS production in bladder tissue identifies a new pathway whereby these channels mediate aging, painful bladder, and overactivity.

### 4.6 Human relevance and pathological significance

The function of TRPV4 in human urothelium has not been experimentally examined previously. An early meeting abstract reported increased bladder contractions in mouse and rat by a non‐specific TRPV4 activator and proposed an interesting idea that TRPV4 contributes to bladder overactivity.^81^ However, this was a simple contraction of normal bladder tissue and bladder overactivity was not substantiated, and no human tissue or pathological overactive bladder was examined. Our study provides evidence that TRPV4 receptors are expressed in native human bladder mucosa with the highest density in the urothelial cells confirming a previous report^39^ and provides the first evidence that these receptors function by augmenting urothelial ATP release—a key urothelial function. Thus, TRPV4 channels are important functional regulators in human bladder with specific action on urothelial function with both sensory and motor activities. The attenuation of spontaneous release of ATP from the human mucosa by a TRPV4 antagonist in the absence of any exogenous activators further demonstrates intrinsic TRPV4 activity in human bladder tissue and the wide functional implications of this receptor in maintaining resting activity of the urothelium and hence normal bladder physiology. The consistent physiological responses and mode of action between human and guinea‐pig preparations provide evidence that TRPV4 receptors in the bladder are universal functional regulators across animals and humans. This specifically identifies a role for TRPV4 in chemically induced and stretch‐evoked urothelial ATP release in physiologically relevant bladder strips and reveals age‐dependent changes that may influence OAB, highlighting the multifactorial potential of TRPV4 to contribute to OAB. Importantly, reduced basal ATP release was achieved with selective blockade of TRPV4, further indicating the potential of this receptor as a drug target.

Importantly, this study provides the first experimental evidence that TRPV4 receptors contribute to greater ATP release in pathological human OAB bladders. This highlights the role of this channel in augmentation of ATP release and the associated downstream sensory and motor up‐regulation in the pathogenesis of bladder overactivity. Furthermore, the data also show for the first time that ageing facilitates TRPV4 activation in OAB urothelium as evidenced by increased proportional ATP release in response to TRPV4 activator. This mode of action is characteristic of pathological OAB bladders but not in non‐OAB bladders where no correlation can be seen, thus uncovering a pathology‐specific mechanism in a human condition.

### 4.7 Summary

In summary, this is the first study to demonstrate a fundamental physiological role of TRPV4 in regulating urothelial ATP release and bladder function across species with human relevance and identifies the mode of action and mechanisms in stretch‐independent responses. Of pathological significance, this study also provides the first evidence for TRPV4 involvement in aging, oxidative stress, and overactive bladders.

## AUTHOR CONTRIBUTIONS

C. Wu, G. Sui, and M. Ruggieri designed research; M.W.G. Roberts, G. Sui, and R. Wu performed research; M.W.G. Roberts, G. Sui, R. Wu, and C. Wu analyzed data; M.W.G. Roberts, G. Sui, and C. Wu wrote the paper; C. Wu and M. Ruggieri made critical revision; W. Rong and S. Wildman contributed to the revision; B. Montgomery, A. Ali, and S. Langley identified the patients and collected human tissues.

## CONFLICT OF INTEREST

The authors have declared that no conflict of interest exists.
